# The first report of co-existence of pulmonary tuberculosis and lung malignancy in a kidney transplant recipient: a case report and literature review

**DOI:** 10.1186/s12879-021-06350-x

**Published:** 2021-07-01

**Authors:** Gang Fang, Ning-Chang Cheng, Li-Li Huang, Wei-Ping Xie, Chun-Mei Hu, Wei Chen

**Affiliations:** 1 Department of Tuberculosis, The Second Hospital of Nanjing, Nanjing University of Chinese Medicine, Nanjing, 210003 China; 2 Department of Respiratory, Xinglong Community Health Center, Nanjing, 210019 China; 3 Department of Respiratory and Critical Care Medicine, Jiangsu Province Hospital, the First Affiliated Hospital of Nanjing Medical University, Nanjing, China; 4 Clinical Research Center, The Second Hospital of Nanjing, Nanjing University of Chinese Medicine, Nanjing, 210003 China

**Keywords:** Kidney transplant recipient, Tuberculosis, Lung cancer, Co-existence

## Abstract

**Background:**

Along with the medical development, organ transplant patients increase dramatically. Since these transplant patients take immunosuppressants for a long term, their immune functions are in a suppressed state, prone to all kinds of opportunistic infections and cancer. However, it is rarely reported that the kidney transplant recipients (KTRs) have pulmonary tuberculosis and lung cancer simultaneously.

**Case presentation:**

A 60-year-old male was admitted because of persistent lung shadow for 2 years without any obvious symptom 8 years after renal transplant. T-SPOT test was positive but other etiological examinations for *Mycobacterium tuberculosis* were negative. Chest CT scan revealed two pulmonary lesions in the right upper and lower lobe respectively. ^18^F-fluorodesoxyglucose positron-emission tomography (FDG-PET) CT found FDG intake increased in both pulmonary consolidation lesions. CT-guided percutaneous transthoracic needle biopsy revealed lung adenocarcinoma and tuberculosis. The video-assisted thoracoscopic surgery was operated to resect the malignancy lesions. The patient received specific anti-tuberculosis therapy and was discharged. At the follow-up of 6 months post drug withdrawal, the patient was recovered very well.

**Conclusions:**

We for the first time reported co-existence of smear-negative pulmonary TB and lung adenocarcinoma in a KTR, which highlighted the clinical awareness of co-occurrence of TB and malignancy after renal transplant and emphasized the value of biopsy and ^18^F-FDG-PET in early diagnosis of TB and cancer.

**Supplementary Information:**

The online version contains supplementary material available at 10.1186/s12879-021-06350-x.

## Background

Kidney transplant is an important surgical procedure to treat kidney failure, improving and prolonging the life of patients. However, successful organ transplant faces a lot of challenges, since many postoperative complications might occur [1, 2]. Among all the infective complications, tuberculosis (TB) is the most common one after renal transplantation. The pooled prevalence of TB included active TB and latent TB infection in kidney transplant recipients (KTRs) is 2.51% worldwide, and is even higher in the developing countries [3, 4]. Post-transplant TB is considered as a major problem in successful long-term outcome of KTRs [5]. Besides TB, cancer is another important post-transplant complication, which is the second cause of mortality and morbidity in KTRs [6]. In 2019, a single center analysis showed that 39 of 535 (7.2%) KTRs developed malignancies within a mean follow-up of 7.8 years [7]. Unfortunately, the prognosis of KTRs with malignancies is not good. Almost half of them die with the average age 58.5 [7].

As we know, KTRs are under immunosuppressed conditions [8]. Even though pre-transplant screening of both donor and recipient for TB and cancer is conducted, reactivation of latent TB, new TB infection or de novo malignancy appear inevitably in some cases. Prompt diagnosis and treatment is pivotal to extend the healthy and productive life of KTRs. However, among different TB types, smear- or culture-negative TB is especially hard to make a definite diagnosis. Additionally, the clinical and radiological features between TB and cancer are very similar, which often leads to misdiagnosis and delayed treatment [9]. Thus, every case with definite diagnosis and different characteristics is valuable for the clinicians. So far, even abundant cases about complicated TB or malignancy among KTRs are already reported, there is no combination of pulmonary TB and lung malignancy in KTRs, which dramatically challenges the success of allograft.

In this case, we for the first time reported co-existence of smear-negative pulmonary TB and lung adenocarcinoma in a KTR, which highlighted the clinical awareness of co-occurrence of TB and malignancy after renal transplant and emphasized the value of biopsy and ^18^F-FDG-PET in early diagnosis of TB and cancer.

## Case presentation

**Fig. 1:**
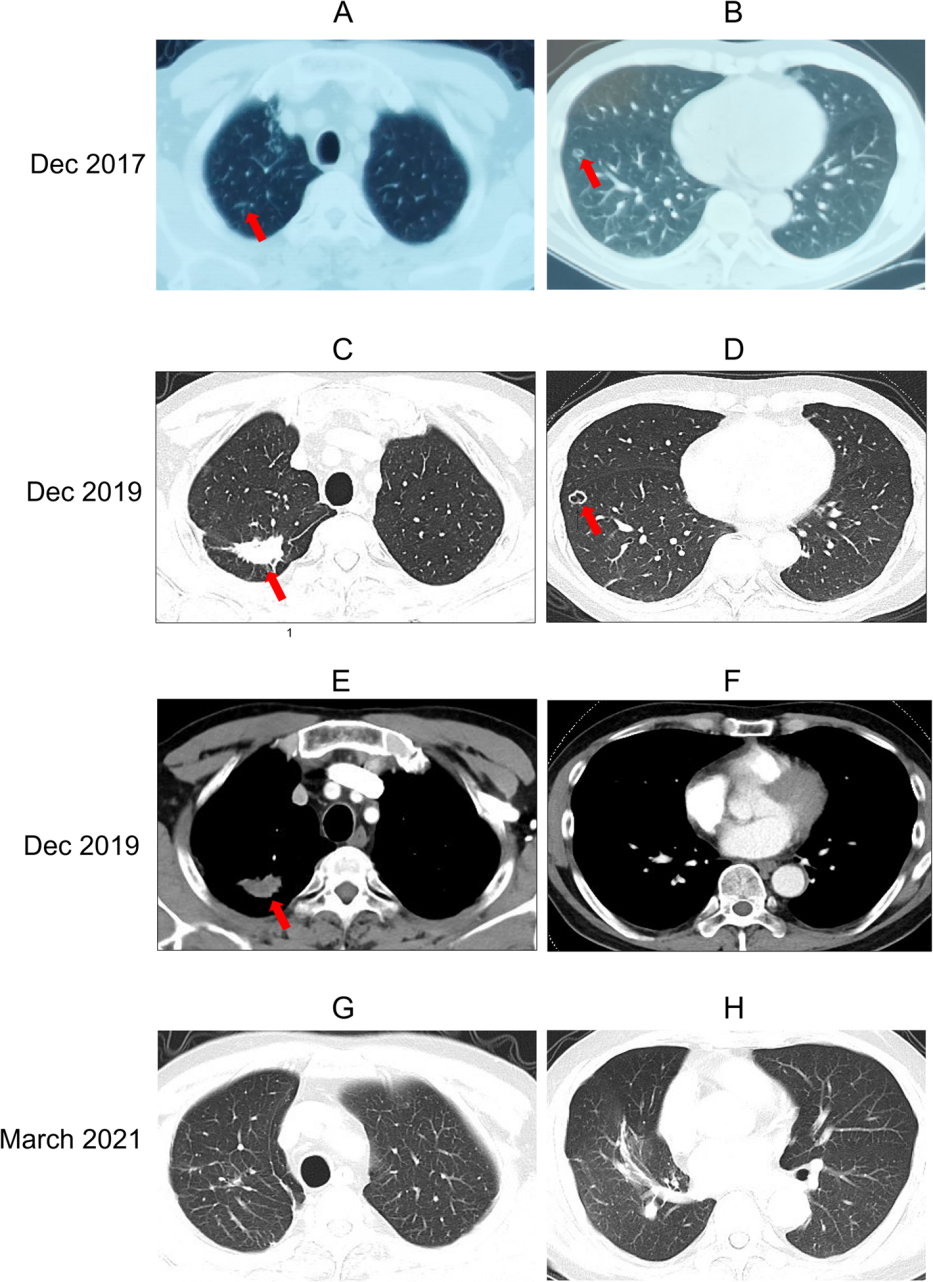
Chest CT at different time points. In December 2017, chest CT revealed speckle and stripe shadows in the posterior segment of the right upper lobe (**A**) and a nodule in the anterior segment of right lower lobe with small cavity shadow inside (**B**). At admission, chest CT found a soft tissue density mass shadow (30 mm × 19.6 mm) with spiculate boundary in the posterior segment of the right upper lobe (**C**) and the nodule (11 mm × 11 mm) in the right lower lobe became bigger than two years ago (**D**). The mass shadow was observed in the mediastinal window (**E**) but not for the nodule (**F**). At the follow-up of 6 months post drug withdrawal, after resection of the original mass, a few cord shadows were seen in the posterior apical segment of the right upper lobe (**H**). After wedge-shaped surgery of the right lower lobe to excise of the original cavitation nodule in the anterior basal segment of the right lower lobe, a few stripe shadows were observed (**G**). Lesions were indicated by red arrows

In December 2019, a 60-year-old man was admitted to the Second Hospital of Nanjing due to persistent lung shadow for 2 years, but without any clinical symptoms. He had received the allograft kidney transplant because of chronic renal failure since 2011. The regular pre-transplant screening reports ever excluded TB or cancer for both the donor and the receipt. Afterwards, the renal function of the patient maintained stable with the combination drug therapy, which was composed of mycophenolate mofetil (500 mg/250 mg per day), cyclosporine (75 mg twice per day), prednisone (5 mg once per day), phoebus, Bailing capsule and valsartan. In December 2017, during the follow-up examination in the kidney transplant hospital, the chest CT found speckle and stripe shadows in the posterior segment of the right upper lobe (Fig. 1A), and a nodule in the anterior segment of right lower lobe with small cavity shadow inside (Fig. 1B). Both T-SPOT and purified protein derivative (PPD) tests were positive for *Mycobacterium tuberculosis* (MTB) infection, but detection of MTB from the sputum samples through smear microscopy, culture and real-time PCR was negative. Then, the empirical anti-TB therapy was started. Whereas, this treatment was terminated after 2 weeks since the patient family worried about indefinite diagnosis of TB and side effects.

At admission, physical examination revealed that respiratory, cardiovascular, abdominal and neurological characteristics were normal. Complete blood count test, C-reaction protein, erythrocyte sedimentation rate, coagulation (D-Dimer), tumor markers, liver and renal function tests were negative. As before, T-SPOT test was positive, but smear microscopy, culture and real-time PCR for MTB were still negative.

**Fig. 2:**
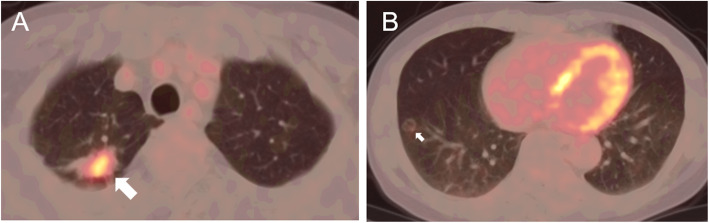
FDG-PET CT. FDG intake increased in both the pulmonary consolidation lesions, with SUVmax 8.2 in the right upper lobe and SUVmax 1.9 in the right lower lobe, respectively. Lesions with increased FDG intake were indicated by arrows

Remarkably, chest CT found some changes compared with 2 years ago. In the posterior segment of the right upper lobe, a soft tissue density mass shadow with spiculate boundary appeared (Fig. 1C). Also, the nodule in the right lower lobe became bigger (Fig. 1D). According to those radiological findings, the possibility of lung cancer in the upper lobe was considered. ^18^F-fluorodesoxyglucose positron-emission tomography (FDG-PET) CT found that FDG intake increased in both the pulmonary consolidation lesions, with SUVmax 8.2 in the right upper lobe and SUVmax 1.9 in the right lower lobe, respectively (Fig. 2). Meanwhile, there was no obvious abnormality for the remaining part of lung, mediastinal lymph nodes, liver and kidney. Taken together, adenocarcinoma was probably considered. A CT-guided percutaneous transthoracic needle biopsy was then operated, and the pathological result confirmed lung adenocarcinoma in the right upper lobe.

**Fig. 3:**
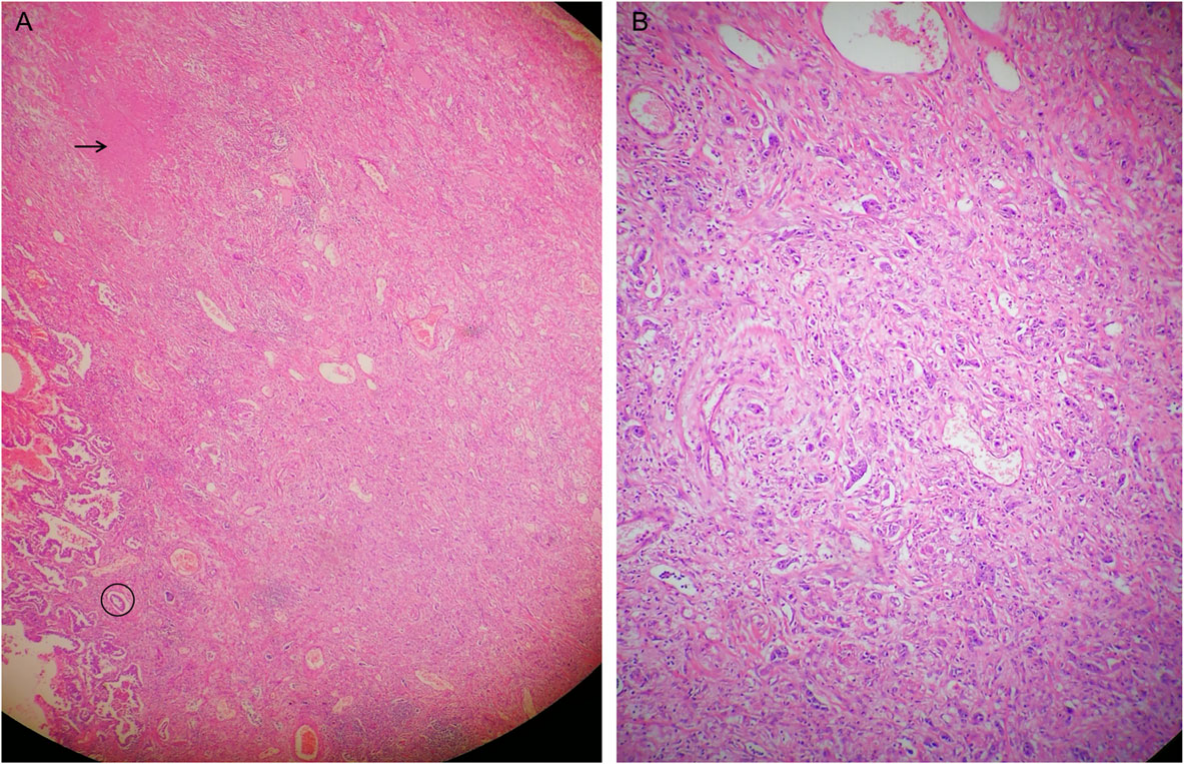
Pathological examination of tissue specimens from two consolidation lesions. **A** The right upper lobe had invasive adenocarcinoma, grade II-III, mainly acinar type (90%), with the size of 2.5 cm × 1.8 cm × 1.5 cm (indicated by black circle). The tumor was peripheral type, which did not break through the pulmonary capsule. Sprinkle multifocal epithelioid granulomatous nodules were seen in the surrounding lung tissues (indicated by black arrow). Caseous necrosis formed along with multinucleated giant cells. **B** The right lower lobe had infiltrating adenocarcinoma, grade III, mainly solid type, with the size of 1.2 cm × 0.9 cm × 0.7 cm. No cancer tissue was found in the lung membrane

**Fig. 4:**
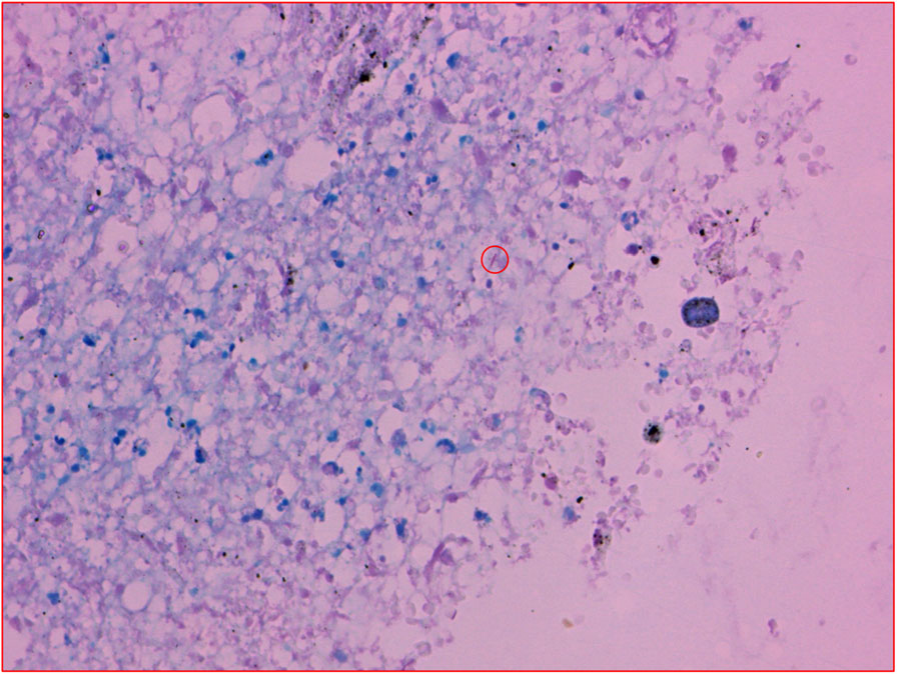
Acid-fast staining to detect MTB for the tissue specimen of the right upper lobe. Only one MTB was observed in the sight field, which was indicated by a red circle

The video-assisted thoracoscopic surgery (VATS) right upper lobectomy, wedge resection of right lower lobe and lymphadenectomy were operated. The pathological results revealed that both lobes had invasive adenocarcinoma but with different histological subtypes, according to the predominant pattern of the neoplastic cell growth, which was mainly acinar type and solid type for the upper lobe and lower lobe, respectively (Fig. 3A and B). To our interest, we observed caseous necrosis along with acinar type adenocarcinoma in the right upper lobe (Fig. 3A). Acid-fast staining for the surgical tissue samples detected MTB in the field (Fig. 4). Real-time PCR was also positive for MTB. Therefore, we could make a definite diagnosis that smear-negative TB and adenocarcinoma co-existed in the right upper lobe.

According to TNM classification criteria, the adenocarcinoma in the right lobe was diagnosed with Stage IA, no chemotherapy or radiotherapy was taken further after surgical resection. Since TB infection was complicated in the right upper lobe, anti-TB treatment was still necessary. However, the challenge for TB treatment was how to avoid potential renal toxicity of anti-TB drugs. Considered that the KTR has been taking anti-rejection medication, rifampicin was firstly excluded because it would affect the expression of cytochrome P450 3A4 in the liver and in the small intestine and interfere with the metabolism of anti-rejection drugs [10]. Similarly, pyrazinamide was also excluded as it would lead to hyperuricemia and influence the renal function. The final anti-TB regimen for this patient was isoniazid (0.3 mg, QD, ethambutol (0.75 mg, QD) and moxifloxacin (0.4 mg, QD). After two-week treatment in hospital, the patient was discharged for continuous anti-TB treatment for 9 months in total.

In March 2021, at 6-month follow-up after drug withdrawal, the patient kept healthy without any uncomfortable symptoms. The normal tests for MTB and tumor were all negative, and the renal function was normal.

## Discussion and conclusion

### Characteristics of KTRs complicated with TB or cancer

We performed literature searching in PubMed database with the keywords: (tuberculosis) AND (lung cancer OR lung carcinoma) AND (kidney transplant OR renal transplant). The search was limited to English literature and other language literature with English abstract. To our best knowledge, this was the first time to report co-existence of pulmonary tuberculosis and lung malignancy in a kidney transplant recipient. However, when using Mesh terms “kidney transplant” & “tuberculosis” (KT/TB) from 2016 to 2020, and finally found 21 cases (Table S1). The KT/TB patients were mainly middle-aged, with the median age 48.29. Gender was not associated with TB incidence. Of 21 KT/TB patients, 10 (47.6%) had none underlying disease. Among the remaining 11 patients, 5 had diabetes and 3 had hypertension. Meanwhile, extra-pulmonary TB was predominant, with 17 of 21 cases (80.95%). The median onset time of TB was 7 months post transplant, with the shortest 3 months and the longest 12 years. Diagnosis of TB mostly depended on culture of MTB and qPCR for MTB DNA through the biopsy samples from lesions*.* RIPE scheme (rifampicin, isoniazid, pyrazinamide and ethambutol) was the most common anti-TB therapy (10/21, 47.62%). The prognosis of KT/TB patients seemed to be optimistic.

As well, the literatures about KT complicated with lung cancer (LC) from 2016 to 2020 were summarized in Table S2. In China, a single-center analysis reported that 0.5% of KTR developed lung cancer from 1988 to 2015 in a southern hospital of China [11]. The mean age of KT/LC patients was 50.2 ± 8.3 years at transplantation. The average interval from renal transplantation to cancer diagnosis was 65.7 ± 20.1 months. In France, a case-control study analyzed retrospectively all KT/LC cases in three French Renal Transplant Units from 2003 to 2012 and found that LC incidence was 1.89/1000 person-years from 2008 to 2012 [12]. The median interval of time from KT to LC diagnosis was 7 years (range 0.5–47 years). In our case, the adenocarcinoma in the right lung might occur 6 years post KT.

### Relationship between TB and cancer

As we know, TB is the major public health threat of human, causing approximately 10 million new infection and 1.2 million death among HIV-negative people worldwide in 2019 [13]. In the meantime, based on the data of “Statista” website, lung cancer has the highest rate of death among different cancer types and causes 23 death per 100,000 population in the world in 2020. Actually, co-existence of TB and lung cancer has been well-described [14, 15]. At one hand, inflammatory process is one of the potential factors of cancer. Pulmonary TB is considered as the most important inflammation-inducing factors in the lungs [16, 17]. Additionally, it’s proposed that TB could cause chromosome breakage of bronchial epithelium and the bacterial DNA could also integrate into the chromosome of cells, leading to neoplastic transformation [15]. At the other hand, lung cancer may cause re-activation or recurrence of latent TB in turn [18, 19]. Immunotherapy of cancer often includes immunosuppressors, predisposing the patients to an immunocompromised state that latent tuberculosis infection is reactivated [20].

Although both the right upper and lower lung were adenocarcinoma, but the specific subtype was different. After kidney transplantation, the patient took immunosuppressant and anti-rejection drugs for a long term. Thus, he was at a status of low immunity and weak autoimmune monitoring, who was prone to develop cancer. In this case, TB was diagnosed earlier and the cancer was at stage IA. We inferred that TB developed first and induced neoplastic transformation. The cancer cells may grow adherently along the acinar duct, resulting in obvious enlargement of the lesion 2 years later. However, the right lower cavity-like nodules develop more slowly in comparison. Because the causes are not exactly the same, the subtypes are different, and ultimately the prognosis and course of the disease are not necessarily the same.

### Challenges on definite diagnosis of TB and lung cancer

Because most individuals presented with atypical and unspecific symptoms and the radio-logical findings between TB and lung cancer were very similar, challenges remain on diagnosing co-existence of tuberculosis and lung cancer [14, 21]. In this case, the presence of cavitary lesion on the chest CT image indicated possible TB infection. Nevertheless, cavities on thoracic images occasionally indicated various infections, autoimmune conditions, or primary and metastatic malignancies [21]. Moreover, the heterogeneous pattern of FDG uptake increase may provide clues to the presence of double pathologies: infection and malignancy [22]. Therefore, CT-guided PTNB was applied for definite diagnosis, which provided high accuracy, sensitivity, and specificity for both malignancy and infection [23]. A recent research revealed that CT-guided PTNB had overall diagnostic accuracy of 83% (19/20) in the evaluation of pulmonary consolidation lesions [24].

### Challenges on anti-TB treatment in KTRs

Anti-TB medications in KTRs caused a high mortality from 12 to 16.7% all over the world [25]. In addition, drug-drug interactions with immunosuppressive drugs might lead to potential renal or liver toxicity [26]. The well-known “first line” anti-TB regimen includes isoniazid, rifampicin, pyrazinamide and ethambutol. In this case, rifampicin was firstly excluded because of its renal toxicity. Pyrazinamide was not used as it would lead to hyperuricemia and influence the renal function. A recent retrospective study proved that concurrent chemotherapy (anti-cancer and anti-TB chemotherapy) was effective and safe for treating cancer patients with active TB [27]. However, immune checkpoint inhibitors such as PD-1/PD-L1 were widely used on cancer treatment, which may induce reactivation of LTBI or develop active TB [28]. Fortunately, this patient only required anti-TB therapy since the cancer lesions were resected by surgery.

In conclusion, this case described a rare phenomenon of co-existence of smear-negative pulmonary TB and lung adenocarcinoma in KTRs, which highlighted the clinical awareness of co-occurrence of TB and de novo malignancy after renal transplant and emphasized the value of biopsy and ^18^F-FDG-PET in early diagnosis of TB and cancer. The anti-TB therapy should consider the complicated drug–drug interactions with immunosuppressive agents, and anti-cancer medications.

## Supplementary Information

**Additional file 1.**

**Additional file 2.**

## Data Availability

Data relating to this study are contained and presented in this document. Other materials are available from the corresponding authors on reasonable request.

## Abbreviation

TB: Tuberculosis
KTR: Kidney transplant recipient
MTB: *Mycobacterium tuberculosis*
CT: Computerized tomography
^18^F-FDG-PET: ^18^F-fluorodeoxyglucose positron-emission tomography
VATS: Video-assisted thoracoscopic surgery
KT: Kidney transplant
LC: Lung cancer
