# Supplementary material for: The first report of co-existence of pulmonary tuberculosis and lung malignancy in a kidney transplant recipient: a case report and literature review
Source: BMC Infect Dis. 2021 Jul 1;21:629. doi: 10.1186/s12879-021-06350-x (PMC8252204; doi:10.1186/s12879-021-06350-x)
Supplement: Supplementary file 1 — Additional file 1. [file 12879_2021_6350_MOESM1_ESM.docx]

## Table S1. Case summary for kidney transplant recipient complicated with tuberculosis worldwide from 2016 to 2020

| **Year** | **Country** | **Age/Sex** | **Underlying**  **Disease** | **Chief Complain** | **Type of TB** | **Onset Infection Time** | **Diagnosis** | **Treatment** | **Prognosis** | **Reference** |
| --- | --- | --- | --- | --- | --- | --- | --- | --- | --- | --- |
| 2020 | México | 40/Male | NA | Abdominal Pain | Extra-pulmonary TB | 4 years post-transplant | An exploratory laparotomy for histopathology specimens and paraganglionic fluid culture | An individualized treatment, positive for rifampicin resistance | NA | [1] |
| 2019 | India | 35/Female | Diabetes | Fever, cough | Multiple subcutaneous abscesses due to TB | 6 months post-transplant | Fine-needle aspiration cytology of the lymph node | RIPE scheme:  rifampicin, isoniazid, pyrazinamide and ethambutol | NA | [2] |
| 2019 | Germany | 63/Male | NA | Acute rise of creatinine | Tuberculous granulomatous interstitial nephritis | 2 months post-transplant | Mycobacterium tuberculosis was  cultured in the urine, blood, and sputum | RIPE scheme | Kidney function improved and remained 5 years later | [3] |
| 2019 | Germany | 51/Female | NA | Epigastric pain, diarrhea, weight loss and night sweats | Disseminated abdominal TB | 10 years post-transplant | Acid stain, polymerase chain reaction (PCR) and culture on colonic mass taken by colonoscopy | Rifampicin, isoniazid and ethambutol | One year later died of pneumonia, all tests and cultures for  TB were negative | [4] |
| 2019 | Portugal | 66/Male | NA | 2 cutaneous lesions localized on the back and abdomen | Cutaneous TB | 6 months post-transplant | Tissue cultures and skin biopsy with histological  analysis, PCR confirmed | Multidrug TB therapy | Lesion clearance after 3 weeks | [5] |
| 2019 | USA | 60/Female | Recurrent pyelonephritis | Fever | Cutaneous gummatous TB | 7 months post-transplant | Peritoneal nodules revealed caseating granulomas and acid-fast bacilli | Anti-TB discontinued due to several thrombocytopenia, and restarted | NA | [6] |
| 2018 | India | 46/Female | Scaly plaques | Zinc deficiency | Gastrointestinal TB | NA | NA | 3 mg/kg/day of elemental zinc and anti-TB therapy | There was complete clearance of the plaques by the end of three months | [7] |
| 2018 | India | 35/Male | NA | Abdominal distension | Constrictive pericarditis due to TB | 4 years post-transplant | NA | Non-rifampicin-based anti-tubercular therapy | Symptomatically improved after 2 months | [8] |
| 2018 | Korea | 65/Female | Hypertension, chronic hepatitis B virus carrier | Cough, paraparesis | Miliary TB | 6 months post-transplant | Acid–fast bacilli (AFB) culture from bronchial alveolar lavage specimens | RIPE scheme | One year after completing disseminated TB, the patient showed no clinical evidence of TB recurrence | [9] |
| 2018 | Russia | 23/Female | Diabetes | Cough, fever | Pulmonary TB | 9 months post-transplant | NA | Robot-assisted lobectomy | After 6 months, the patient had no complains and PCR negative for TB | [10] |
| 2018 | India | 26/Female | NA | Tiny hard swelling over dorsum of left foot and right thumb | Multi-focal tuberculous osteomyelitis | 3 months post-transplant | Fine needle aspiration of pus | RIPE scheme | NA | [11] |
| 2018 | India | 35/Female | Abdominal TB | Dyspnea on exertion and cough with expectoration | Tuberculous interstitial nephritis | 3 months post-transplant | Chest X-ray | Subcapsular graft nephrectomy | NA | [12] |
| 2017 | Slovakia | 53/Female | Hypertension | Septic shock, multiple organ failure | Gastrointestinal TB | 7 years post-transplant | Histology tissue specimens obtained from the abdominal cavity | RIPE scheme | After 34 days, the patient was discharged, followed by complete recovery. | [13] |
| 2017 | Portugal | 62/Male | NA | Polaquiuria, dysuria and fatigue | Genitourinary TB | 15 months post-transplant | Prostatic biopsy | RIPE scheme | The patient was discharged and follow-up was  maintained in our outpatient unit | [14] |
| 2017 | Brasil | 56/Female | NA | Papules and ulcers in the right forearm | Cutaneous and articular TB | 7 months post-transplant | Asynovial fluid  culture | Surgical fistulectomy and anti-TB therapy | After 12 months, the patient cured | [15] |
| 2017 | India | 22/Female | NA | Intractable cough | Tuberculous bronchoesophageal fistula | 9 years post-transplant | Sputum was positive for  AFB | Thoracotomy and closure of rents was done along with lobectomy of right middle and lower lobes | NA | [16] |
| 2017 | Tunisia | 25/Male | NA | Fever, asthenia, abdominal pain, and myalgia | Lymph node TB | 6 months post-transplant | Bacteriologic examination of the drainage liquid | RIPE scheme along with ciprofloxacin and piperacillin sodium/tazobactm sodium | At 5-year follow-up, the patient was still doing well after TB  infection | [17] |
| 2017 | Nepal | 66/NA | Hypertension | NA | Pulmonary TB | 11 months post-transplant | NA | Antibiotic, stereotactic aspiration and anti-TB therapy | NA | [18] |
| 2017 | India | 57/Male | Diabetes | Dysphagia, odynophagia, retrosternal heaviness, hiccough, and vomiting | Esophageal TB | NA | Histopathological examination (AFB) on multiple biopsy samples taken from the esophageal lesions | RIPE scheme along with valacyclovir and  fluconazole | At a follow up of 5 months, the patient was asymptomatic and had excellent graft function | [19] |
| 2016 | USA | 74/Female | Diabetes | Fever | Disseminated TB | 4 months post-transplant | Nucleic acid amplification testing | RIPE scheme | NA | [20] |
| 2016 | Iraq | 54/Male | Diabetes, hypertension | Fever, malaise and confusion | Multiple intracranial TB | 6.5 years post-transplant | Diffuse and bilateral ring enhancement cerebral lesions on MRI | RIPE scheme | The patient was treated successfully | [21] |

**References:**

[1]. Hernandez-Robles, C.M., et al., Forty-Year-Old Man With Abdominal Pain 4 Years Post-Renal Transplant: A Case Report. Transplant Proc, 2020. 52(4): p. 1206-1208.

[2]. Khandalvalli, P., S. Nazneen and M. Yadla, An unusual clinical presentation of tuberculous pyomyositis in a renal allograft recipient. Saudi J Kidney Dis Transpl, 2019. 30(5): p. 1175-1178.

[3]. Schmidt-Lauber, C., et al., Tuberculous granulomatous interstitial nephritis in a renal allograft. Kidney Int, 2019. 96(5): p. 1243.

[4]. Schmidt-Lauber, C., et al., Suspected colonic cancer turns out to be disseminated tuberculosis in a kidney transplant recipient: A case report. Medicine (Baltimore), 2019. 98(36): p. e16995.

[5]. Coelho, I.D., et al., A Rare Manifestation of Tuberculosis in a Renal Transplant Patient: A Case Report. Transplant Proc, 2019. 51(5): p. 1618-1620.

[6]. Evans, N.S., et al., Cutaneous gummatous tuberculosis in a kidney transplant patient. Cutis, 2019. 103(2): p. E32-E35.

[7]. Ghuge, P., R. Karia and R.H. Malkani, Acquired zinc deficiency in a renal transplant recipient with gastrointestinal tuberculosis responding promptly to oral correction. Saudi J Kidney Dis Transpl, 2018. 29(5): p. 1199-1202.

[8]. Kasinadhuni, G., et al., A rare cause of ascites in a renal transplant recipient. BMJ Case Rep, 2018. 2018.

[9]. Kim, Y., S.P. Kim and S. Han, Multiple tuberculomas invading the central nervous system as a paradoxical reaction in a kidney transplantation recipient. Saudi J Kidney Dis Transpl, 2018. 29(3): p. 719-722.

[10]. Yablonskii, P.K., et al., Robot-assisted lobectomy for pulmonary tuberculosis in a case with immunosuppression after kidney transplantation. Int J Tuberc Lung Dis, 2018. 22(6): p. 704-705.

[11]. Padmavathi, D.S., et al., Multi-focal tuberculous osteomyelitis in a kidney transplant patient. Nephrology (Carlton), 2018. 23(3): p. 288-289.

[12]. Padmanabhan, A., et al., Acute cellular rejection with coexisting tuberculous interstitial nephritis in renal allograft. Saudi J Kidney Dis Transpl, 2018. 29(1): p. 214-216.

[13]. Cikova, A., et al., Gastrointestinal tuberculosis following renal transplantation accompanied with septic shock and acute respiratory distress syndrome: a survival case presentation. BMC Gastroenterol, 2017. 17(1): p. 131.

[14]. Rodrigues, N.J., et al., Genitourinary tuberculosis - a rare presentation of a still frequent infection in renal transplant recipients. J Bras Nefrol, 2017. 39(2): p. 224-228.

[15]. Moyses-Neto, M., et al., Cutaneous and articular tuberculosis in a renal transplant recipient. Rev Soc Bras Med Trop, 2017. 50(4): p. 565-567.

[16]. Indiran, V., Tuberculous bronchoesophageal fistula presenting as intractable cough. Tuberk Toraks, 2017. 65(1): p. 60-62.

[17]. Barbouch, S., et al., Tuberculosis After Renal Transplant. Exp Clin Transplant, 2017. 15(Suppl 1): p. 200-203.

[18]. Khadka, P., et al., Disseminated Nocardiosis in renal transplant recipient under therapy for pulmonary tuberculosis: a case report. BMC Res Notes, 2017. 10(1): p. 83.

[19]. Kumar, S., et al., Esophageal tuberculosis with coexisting opportunistic infections in a renal allograft transplant recipient. Transpl Infect Dis, 2017. 19(1).

[20]. Baghban, A., et al., Disseminated Mycobacterium tuberculosis following renal transplant with alemtuzumab induction. BMJ Case Rep, 2016. 2016.

[21]. Yadegarynia, D., et al., Multiple intracranial tuberculomas in a post-kidney transplant patient. Saudi J Kidney Dis Transpl, 2016. 27(1): p. 135-8.
