# Supplementary material for: The first report of co-existence of pulmonary tuberculosis and lung malignancy in a kidney transplant recipient: a case report and literature review
Source: BMC Infect Dis. 2021 Jul 1;21:629. doi: 10.1186/s12879-021-06350-x (PMC8252204; doi:10.1186/s12879-021-06350-x)
Supplement: Supplementary file 2 — Additional file 2. [file 12879_2021_6350_MOESM2_ESM.docx]

Table S2. Cases of kidney transplant complicated with lung cancer worldwide from 2016 to 2020.

| **Year** | **Country** | **Age/Sex** | **Underlying Disease** | **Chief Complain** | **Type of Lung cancer** | **Onset Time** | **Diagnosis** | **Treatment** | **Prognosis** | **Reference** |
| --- | --- | --- | --- | --- | --- | --- | --- | --- | --- | --- |
| 2018 | Australia | 52/Male | Hypertension | Right lower limb oedema | Primary lung malignancy | 1 week post-transplant | Bone marrow trephine biopsy, cells were positive for thyroid transcription factor 1 supporting primary lung malignancy | The patient was unsuitable for chemotherapy due to his poor performance | He was dead a week after the diagnosis | [1] |
| 2017 | China | 50.2±8.3 years (14 patients) | Smoking (71.4%, 10/14 patients) | NA | NA | The average interval from transplantation to cancer diagnosis was 65.7±20.1 months. | The malignancies were detected incidentally and 5  patients were symptomatic | 10 patients (71.4%) received surgical treatment | 11 patients died from cancer metastasis or  organ failure and 3 remained alive with functioning graft. The overall 5-year survival rate after diagnosis was 17.9% in these 14 patients. | [2] |
| 2017 | French | Median age 60 years, range 29-85, male/ female ratio  80/20% | All patients were former or active smokers (median 30 pack-years) | NA | Most patients (70%) had advanced lung cancer (stage III or IV) disease. | The median interval of time from kidney transplantation to diagnosis of lung cancer was 7 years (range 0.5-47 years) | Lung cancer was incidentally  diagnosed in 40%. | Surgery and  chemotherapy were proposed to the same proportion of patients. | In cases, mortality was cancer related in 87% and median survival time after diagnosis was  24 months | [3] |
| 2016 | USA | 74/Male | Diabetes, hypertension | NA | Stage IV squamous non-small cell lung cancer | 3 years pre-transplant | NA | Nivolumab | NA | [4] |

**References:**

[1]. Vilayur, E., et al., Metastatic lung adenocarcinoma- associated thrombotic microangiopathy in a renal transplant recipient. BMJ Case Rep, 2018. 11(1).

[2]. Zhang, S.X. and Y. Liu, Primary lung cancer in Chinese renal transplant recipients: a single-center analysis. Nan Fang Yi Ke Da Xue Xue Bao, 2017. 37(6): p. 715-720.

[3]. Rousseau-Gazaniol, C., et al., Lung cancer in renal transplant recipients: A case-control study. Lung Cancer, 2017. 111: p. 96-100.

[4]. Boils, C.L., D.N. Aljadir and A.W. Cantafio, Use of the PD-1 Pathway Inhibitor Nivolumab in a Renal Transplant Patient With Malignancy. Am J Transplant, 2016. 16(8): p. 2496-7.
